# Microparticles produced by human papillomavirus type 16 E7-expressing cells impair antigen presenting cell function and the cytotoxic T cell response

**DOI:** 10.1038/s41598-018-20779-2

**Published:** 2018-02-05

**Authors:** J. Zhang, C. Burn, K. Young, M. Wilson, K. Ly, M. Budhwani, A. Tschirley, A. Braithwaite, M. Baird, M. Hibma

**Affiliations:** Department of Pathology, Dunedin School of Medicine, University of Otago, Dunedin, New Zealand

## Abstract

High-risk, cancer-causing human papillomaviruses (HPV) cause infections of the epidermis that may progress to cancer, including cervical cancer. Viral persistence, contributed to by viral evasion of the host immune response, is associated with the likelihood of cancer developing. Langerhans cells (LCs) are the only professional antigen presenting cells located in the epidermis, therefore may influence the antiviral immune response. Microparticles, or microvesicles, are small membrane particles shed by cells that can exert effects on other cells at both a local and systemic level. We found increased numbers of microparticles were shed from human or mouse keratinocytes expressing the HPV16 E7 oncoprotein, compared with control keratinocytes. Co-culture of LCs with microparticles from E7-expressing cells suppressed the cytotoxic T cell response. We attributed this, at least in part, to the reduction in surface of CD40 and intracellular pro-inflammatory cytokine IL-12 p40 subunit that we measured in the LCs. The evidence provided here shows that co-culture of E7-microparticles with LCs inhibits antigen-specific cytotoxicity. This is an important finding, suggesting that microparticles from HPV-infected cells could suppress the T cell response by regulating LCs, potentially contributing to persistence of HPV infection and cancer.

## Introduction

Human papillomavirus type 16 (HPV16) is a cancer-causing virus that can persist, increasing the probability of malignant transformation of cells^1^. HPV is responsible for almost half of all virally-induced cancers, and is causally associated with greater than 99% of cervical cancer cases^2^. There are fourteen oncogenic, ‘high-risk’ cancer-causing HPV types^3^. One of those high-risk types, HPV16, is responsible for over 50% of cervical cancer cases^3^. High-risk HPV types express oncogenic E6 and E7 proteins, and their expression is necessary for malignant transformation of infected cells to occur^4^.

Around 70% of HPV lesions of the cervix eventually regress by 24-months post-infection. A primary mediator of immune-mediated viral clearance is the CD8+ T cell response. CD8+ T cells are considered highly effective against intracellular pathogens such as viruses, binding to and lysing infected cells, and secreting IFNγ, which has a range of anti-viral effects^5^. In animal models of papillomavirus infection regression is associated with infiltration of CD8+ and CD4+ T cells^6^, and in humans there is a higher frequency of CD8+ T cells in CIN2/3 HPV lesions that regress^7^, suggesting that CD8+ T cells have a direct role in clearance of HPV.

Activation of cytotoxic T lymphocytes (CTLs) requires antigen presenting cell (APC) engagement via MHC complexed with processed peptide, concurrent co-stimulatory molecule binding and cytokine secretion, particularly IL-12, by the APCs^8,9^. In the case of HPV infection, the only APCs that are at the infection site are Langerhans cells (LCs). LCs form a contiguous network within the epidermal layer of the skin^10^. Seneschal, *et al*.^11^ have shown that LCs in the epidermis are influenced by the microenvironment, having the capacity to regulate the phenotype of the skin resident memory T cells (T_RM_) to an effector phenotype for viral clearance, or a suppressor phenotype that may be permissive for viral persistence.

Around 30% of HPV lesions persist or advance to higher-grade lesions, with a greater potential to progress to cancer^12,13^. There is evidence of viral impairment of the CD8 T cell response, and we reported that E7 co-expression in murine basal keratinocytes *in vivo* suppresses the T cell response to ovalbumin (OVA). The contribution of LCs to this remains unclear in that the systemic suppression of T cells in the mouse also occurred following their depletion. However, in HPV-infected epidermis Matthews *et al*. and others showed a quantitative reduction in LCs^14,15^, suggesting viral regulation of LC migratory function. LCs normally are retained in the epidermis by homotypic adhesion between E-cadherin molecules on the LCs and neighbouring keratinocytes. The reduction in LC density was considered to be at least in part due to virally-mediated suppression of E-cadherin expression on the infected keratinocytes^16^. It is not clear whether those LCs remaining at the site of infection are functionally impaired, and if so the mechanisms by which that might be mediated by the neighbouring infected keratinocytes, as the LCs themselves are not permissive for HPV replication.

Cells release various membrane vesicles, including microparticles and exosomes, into the extracellular environment. Cellular microparticles are a heterogeneous group of small membrane fragments, 0.1–1 µm in diameter^17^, also known as secreted microvesicles or ectosomes. They are collectively defined by their size and the surface expression of phosphatidylserine (PS)^18^. Microparticles comprise of a phospholipid bilayer surrounding cytosolic contents, including proteins and mRNA, from the cell from which they are derived^19^. Eukaryotic cells produce microparticles under normal conditions, and following cellular stress^20^. They are able to exert effects on “target” cells locally, regionally or systemically, providing a mechanism of cellular communication that can act at a site distant from the origin of the signal^21^. Microparticles have been implicated in the transfer of proteins and receptors between cells, cell-to-cell communication, cancers and the modulation of immunity and inflammation^22^.

Based on our previous observations that HPV16 E7 expression in cells alters LC and T cell function, and on the reported immune modulatory effects of MPs, we hypothesise that microparticles shed from cells expressing HPV16 E7 have an immune regulatory function on neighbouring LCs. The purpose of this study was to determine the effect of HPV16 E7 expression on microparticle production by cells, and establish the effects of microparticles from E7 expressing cells on the migration and activation capacity of LCs on T cells, and consequentially, their function.

## Results

### Expression of HPV16 E7 increases microparticle release from mouse and human immortalized keratinocytes

**Figure 1:**
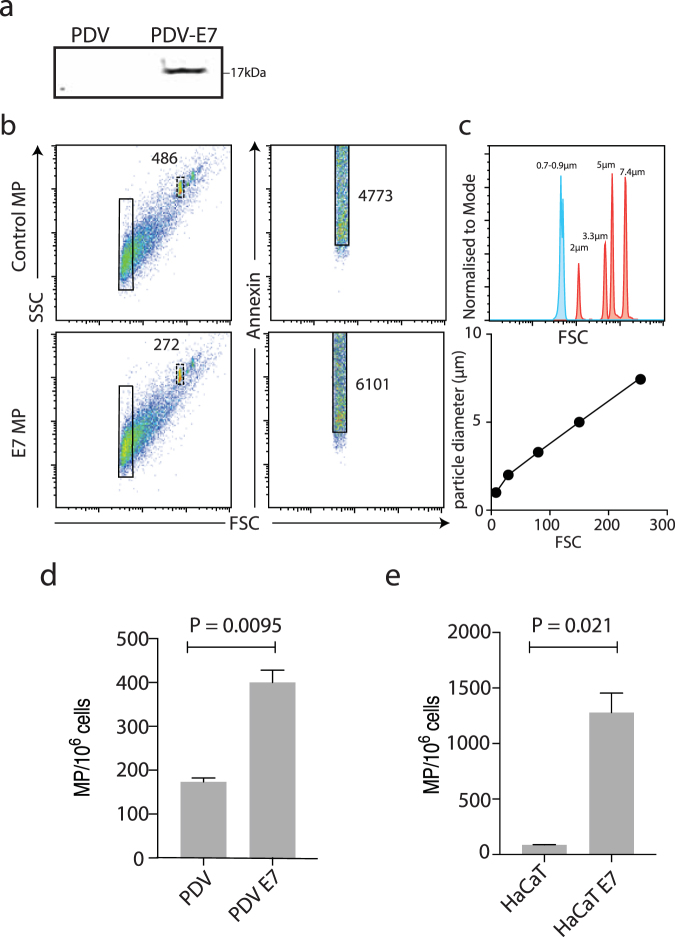
Flow cytometric analysis and quantification of microparticles. (**a**) Western blot of lysates prepared from E7 and control E7rev PDV cells, using an antibody to HA. (**b**) The microparticle population was defined by size based on the use of sizing beads is presented on a log scale histogram, and graphed linearly (**c**). The left-hand panels show the forward and size scatter gating of the microparticles. The absolute number of microparticles was determined using a defined number of 5.2 µm counting beads, indicated in the upper right box in the forward and side scatter plots. The right-hand panels indicate the annexin positive events (i.e. phosphatidylserine positive events), with the gate set using unstained control samples. Quantification of microparticles from (**d**) PDV and (**e**) HaCaT control and E7-expressing mouse keratinocytes (n = 3 independent experiments; mean ± standard error of the mean (SEM) is shown; Welch’s t-test).

We wished to determine if microparticles were shed from E7-expressing and control keratinocytes. In order to test this, several cell lines were generated. Mouse immortalized keratinocytes (PDV cells) expressing a HA-tagged HPV16 E7 under the EF-1 promoter, or a control construct with E7 cloned in reverse E7rev PDV, were produced by transduction of PDV cells with lentivirus and selection with puromycin. Expression of E7 in the selected E7 but not the E7rev PDV cells was confirmed by western blot (Fig. 1a).

In order to establish if microparticles were produced from the E7 and E7rev PDV cells, equal numbers of cells were cultured O/N, and the microparticle containing fraction purified using standard protocols^23^. The samples were stained with PS, mixed with 5.2 µm counting beads and analysed by flow cytometry (Fig. 1b). A size gate was set on the forward/sidescatter plot based on analysis of sizing beads ranging from 0.7–7.4 µm (Fig. 1c). Both the E7 and E7rev cell lines produced PS positive events in the size range consistent with them being microparticles. Interestingly, there were significantly more microparticles produced by the E7-expressing PDV cells (Fig. 1d) when compared with the control E7rev cells. The number of microparticles produced by the transduced control cells did not differ significantly from untransduced cells (data not shown).

**Figure 2:**
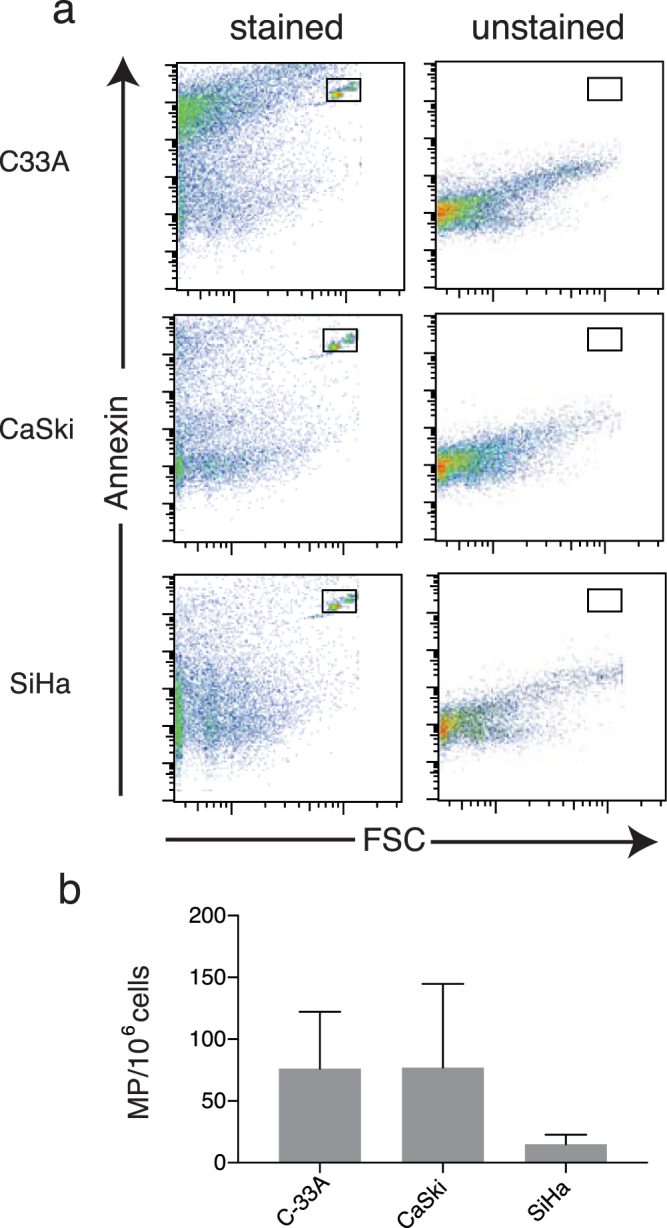
Flow cytometric analysis and quantification of microparticles from human cell lines. (**a**) The microparticle gate was defined using the 0.7–0.9 µm sizing beads. The left-hand panels show the forward and size scatter gating of the microparticles. The absolute number of microparticles was determined using a defined number of 5.2 µm counting beads, indicated in the upper right box in the forward and side scatter plots. The right-hand panels indicate the annexin positive events (i.e. phosphatidylserine positive events), with the gate set using unstained control samples. Quantification of microparticles from (**b**) C-33A, CaSki and SiHa cells is shown (n = 3 independent experiments; mean ± standard error of the mean (SEM); A two-way ANOVA, not significant).

To test if this was also the case for human cells, a human immortalized keratinocyte cell line (HaCaT) was similarly transduced and E7 and E7rev cells selected. Consistent with what was observed in the mouse keratinocytes, there was a significant increase in the number of microparticles released by the human E7-expressing keratinocytes after O/N culture of the cells, when compared with the matched control cell line (Fig. 1e; Fig. S1). We then looked at cells derived from human cervical tumours. CaSki cells are HPV16 E6/E7 positive cells derived from cervical cancer that metastasized to the small intestine^24^, SiHa are HPV16 E6/E7 positive cells from a grade II squamous cell carcinoma^25^, and C-33A cells are HPV negative cells originally obtained from a cervical carcinoma^26^. CaSki and C-33A cells produced similar numbers of microparticles, whereas SiHa consistently produced fewer microparticles than the other two cell lines (Fig. 2). We also tested the effects of HPV16 E6 on microparticle expression in PDV cells and found that similar to E7, E6 increased the numbers of microparticles being produced by these cells (S3). From these experiments we conclude that in addition to E7 E6 expression also increases the number of microparticles produced by PDV cells, and propose that other as yet unidentified  genes also contribute to regulation of microparticle release in CaSki, SiHa and C-33A cells.

### E-cadherin expression on LCs following LPS activation is not altered by co-incubation with E7 microparticles

**Figure 3:**
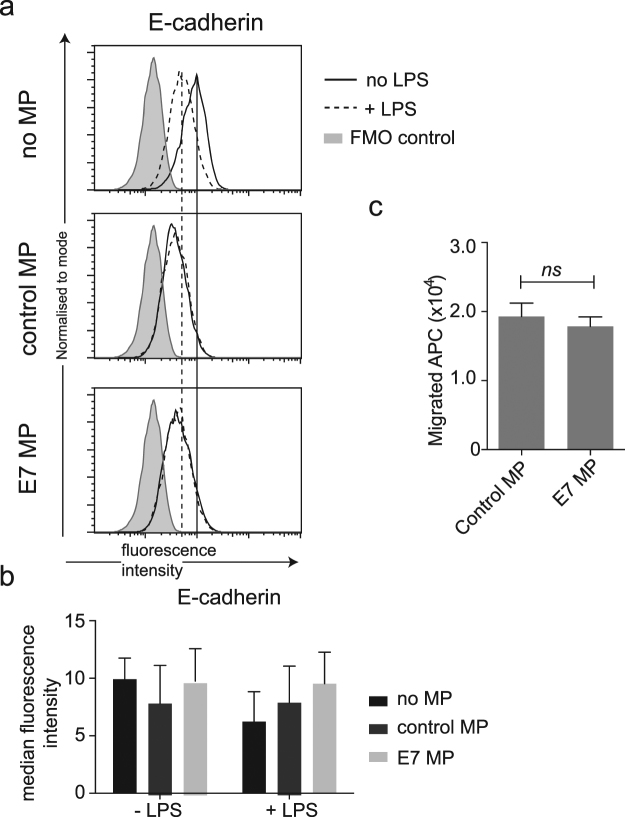
E-cadherin expression and APC migration in response to microparticles produced by E7 expressing cells. LCs were divided into three groups and treated for 48 h with E7 microparticles (E7-MP), control microparticles (control MP) or without MP treatment. Half of the cells in each group were further stimulated with 100 ng/ml LPS for 24 h prior to flow cytometry analysis. (**a**) Histograms of representative samples stained for E-cadherin are shown. Vertical lines indicate the median fluorescence intensity of the no microparticle control LPS treated and untreated samples. (**b**) The median fluorescence intensity for each of the treatment groups is shown. Data shown are the mean ± SEM of 3 independent experimental replicates. Two-way ANOVA following Sidak’s multiple comparisons test was used in the statistical analysis. There was no significant difference between any of the groups. (**c**) Dorsal halves of mouse ears were cultured in the presence of equivalent numbers of E7 or control microparticles for 36 h. The mean (±SEM) number of APC that migrated out of the ears was ascertained by counting using a haemocytometer. Data shown are the results from two experiments completed in triplicate or quadruplicate. Statistical significance was determined by Welch’s t-test. ns: not significant; *P* > 0.05.

E-cadherin is an adhesion molecule expressed on LCs and keratinocytes. It has previously been reported that E-cadherin is reduced on HPV infected keratinocytes in human tissue, and on HPV E6 or E7 expressing keratinocytes *in vitro*. E-cadherin expression is also typically reduced on activation of LC, and its loss is associated with LC migration from the skin. We questioned whether exposure of LCs to microparticles from E7-expressing keratinocytes might further reduce E-cadherin expression on LCs, potentially contributing to their sustained reduction in number in HPV-infected lesions. To test this, LCs differentiated from mouse bone-marrow^27^ (Fig. S2) were co-cultured with microparticles from E7 or control cells then activated with the TLR4 ligand lipopolysaccharide (LPS), and surface E-cadherin expression was measured using flow cytometry. As predicted, there was a reduction in E-cadherin expression following LPS activation in the absence of microparticles. In the presence of E7rev microparticles E-cadherin expression did not alter significantly following LPS treatment, and there was no difference in expression of E-cadherin on LCs when cultured with microparticles from E7 expressing cells when compared to the E7rev microparticles (Fig. 3).

### Migration of LCs from skin explants occurs similarly in the presence of E7 and control microparticles

We questioned whether the reduction in the number of LCs in HPV16-infected skin may be due to increased LC migration as a result of exposure to microparticles expressing E7, irrespective of the lack of difference in E-cadherin expression. To test this, murine dorsal ear skin explants were cultured for 48 h either in Iscove’s medium alone or in medium in the presence of microparticles. Under those conditions, LCs spontaneously migrate from the skin into the tissue culture medium^28^. We found that migrated LCs were detected in the culture media by 48 h when co-cultured with E7rev microparticles. However incubation of explants with equivalent numbers of microparticles from E7-expressing PDV cells did not significantly alter the number of LCs that migrated from the skin (Fig. 3c). From this we concluded that E7 microparticles did not alter the ability of LCs to migrate from the skin within the first 48 h of culture.

### Microparticles from HPV16 E7 expressing cells suppress the cytotoxic T cell response

**Figure 4:**
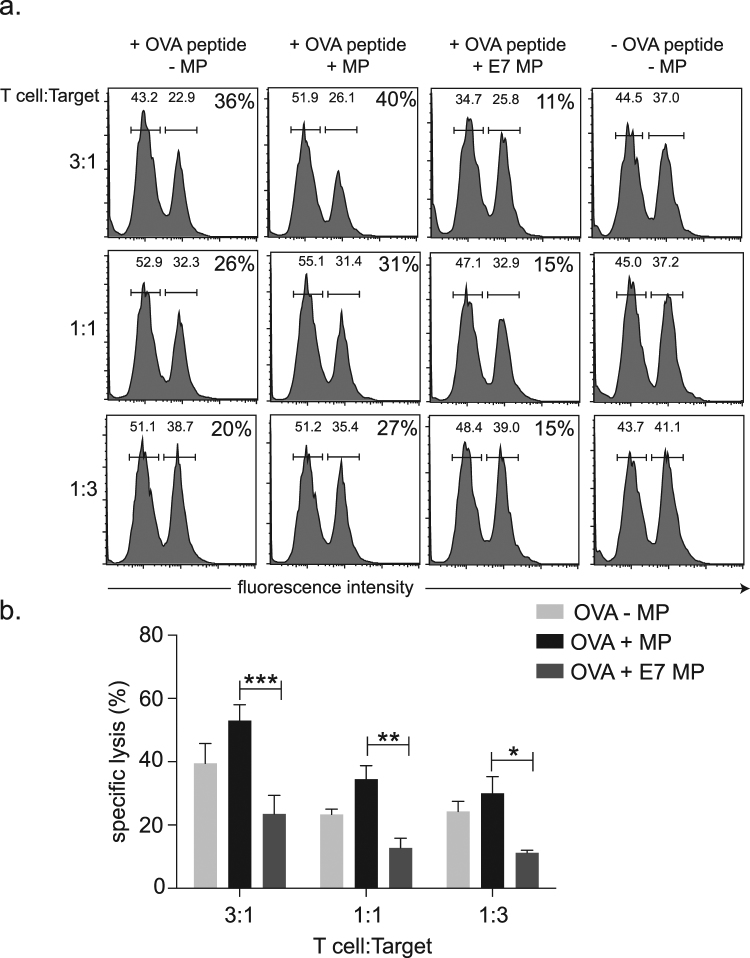
The effects of E7 and control microparticles on the cytotoxic T cell response to OVA pulsed target cells *in vitro*. (**a**) LCs were incubated without microparticles (−MP), with microparticles from control cells (+MP), or microparticles from HPV16E7 expressing cells (+E7 MP) prior to incubation with OVA and OT-I CD8+ T cells (+OVA peptide). CD8 T cells primed by non-OVA bearing LCs were used as the controls (-OVA peptide). OVA-pulsed, CFSE strong splenocytes and OVA-free CFSE weak splenocytes were incubated with the primed OT-I CD8+ T cells at the T cell:target ratios indicated. Representative histograms are shown for each of the treatment groups, indicating the strong and weakly stained CFSE peaks, and with the peptide specific cytotoxicity (% specific lysis) indicated in the top right of each histogram. (**b**) The mean percentage specific lysis for each of the ratios and treatment groups as indicated. Data is the mean of four independent experiments ± SEM. A two-way ANOVA followed by Sidak’s multiple comparisons test was used for the statistical analysis. *P < 0.05; **P < 0.01; ***P < 0.001.

We have previously reported systemic suppressive effects on the T cell response by E7 expressed in mouse keratinocytes. Here we hypothesize that the microparticles produced by HPV16 E7-expressing keratinocytes may contribute to the suppression of the cytotoxic T cell response. To test this, bone marrow differentiated LCs were incubated with the H-2Kb-restricted SIINFEKL peptide from ovalbumin (OVA), and co-cultured with equivalent numbers of E7 or control microparticles and CD8 T cells from the SIINFEKL-specific T cell receptor expressing OT-1 mice. The cytotoxic response generated against OVA peptide-coated targets by T cells primed with LCs was then measured. We found that incubation with E7 microparticles significantly reduced CD8 T cell cytotoxicity for the OVA specific targets by more than half when compared with control microparticles (Fig. 4). From this we concluded that E7 microparticles inhibited the generation of CD8 effector cells against OVA peptide.

### CD40, but not CD86, expression is reduced on LCs co-cultured with HPV16 E7 microparticles

**Figure 5:**
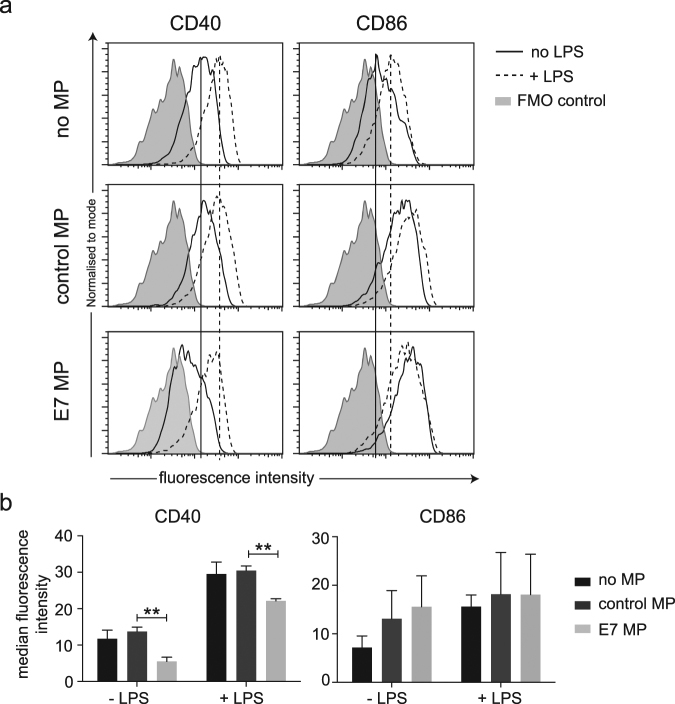
The effect of E7 and control microparticles on co-stimulatory molecule expression on LPS-treated LCs. LCs were divided into three groups and were treated for 48 h with E7 microparticles (E7-MP), microparticles produced from control E7rev PDV cells (control MP) or with no MP treatment. Half of the cells in each group were then further stimulated with LPS, 24 h prior to flow cytometric analysis. (**a**) Histograms showing representative samples stained for CD40 (left column) and CD86 (right column). Vertical lines indicate the median fluorescence intensity of the no microparticle control treated and untreated samples. (**b**) The median fluorescence intensity for each of the treatment groups is shown. Data shown are the mean ± SEM of three independent experimental replicates. Two-way ANOVA following Sidak’s multiple comparisons test was used in the statistical analysis. **P < 0.01.

We questioned if the reduced cytotoxic T cell response may be a result of E7-microparticle inhibition of one or more of the signals delivered by LCs during antigen presentation. Firstly we examined CD40, which engages through CD40L on T cells to license the APC to prime T cells, and induces IL-12 production by LCs^29^. To elucidate if there was an inhibitory effect of E7 microparticles on CD40 expression by LCs they were co-cultured with microparticles from E7 or control cells then activated with LPS, and surface molecule expression was measured using flow cytometry. We found that expression of CD40 was reduced by around 50% following co-culture of cells with E7 but not E7rev microparticles (Fig. 5). Following activation of LCs with LPS, expression of CD40 remained significantly reduced compared with expression on LCs incubated with E7rev microparticles.

Expression of CD86, a co-stimulatory molecule that when engaged by CD28 on T cells lowers the threshold for their activation, was also examined^30^. There was evidence of increased CD86 expression as a result LPS treatment of cells, however CD86 expression was also increased following the addition of microparticles, suggesting that the microparticles stimulated CD86 expression in the absence of LPS treatment. However there was no significant difference in CD86 expression on LCs as a consequence of E7 microparticle co-incubation, compared with control microparticles, following LPS activation (Fig. 5).

### IL-12 p40, but not IL-10, secretion by LCs following LPS activation is inhibited following co-culture with E7-microparticles

**Figure 6:**
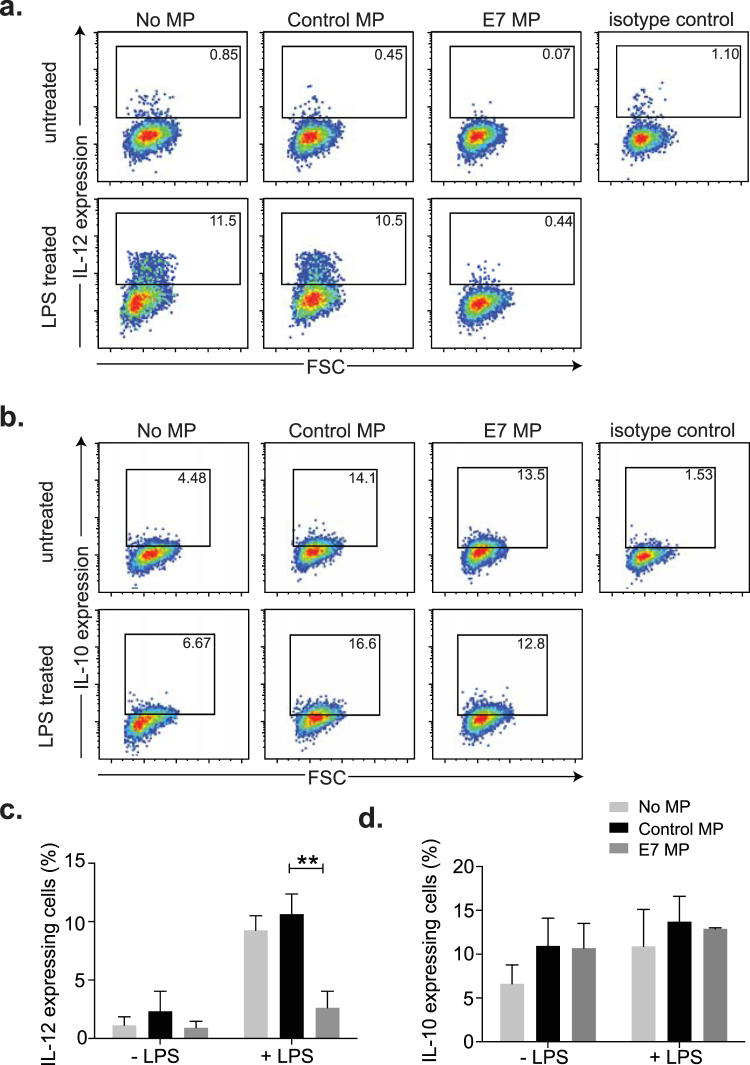
The effect of E7 and control microparticles on cytokine production by LPS-treated LCs. LCs were divided into three groups and treated for 48 h with E7 microparticles (E7-MP), control microparticles (control MP) or without MP treatment. Half of the cells in each group were further stimulated with 100 ng/ml LPS for 24 h prior to flow cytometry analysis. Dot plots indicating intracellular staining for IL-12 (**a**) and IL-10 (**b**) is shown. The percentage of positive gated events is indicated in the top right of each gate. The percentage positive events for IL-12 (**c**) and IL-10 (**d**) are represented graphically. Data is the mean ± SEM of three independent experiments. Two-way ANOVA followed by Sidak’s multiple comparisons test was used for the statistical analysis. **P < 0.01.

IL-12 is a pro-inflammatory cytokine that is regarded as the third signal delivered by antigen presenting cells that is required for the induction of CD8 T effector cells with optimal cytotoxic activity. LPS upregulates the production of IL-12 by LCs, by stimulation through TLR4. LPS was used here to determine the effect of MP from E7 or control cells, and we measured intracellular expression of the p40 subunit of IL-12 by LCs. As expected, IL-12 p40 positive LCs were readily detected by flow cytometry following incubation with LPS alone and intracellular staining. Co-incubation of cells with the MP preparation from control cells did not significantly alter the percentage of IL-12 p40 expressing LCs (Fig. 6). In contrast, IL-12 p40 expression was ablated when cells were incubated with E7-expressing MP and LPS. Indeed the level of suppression was such that the percentage of IL-12 p40 positive cells was comparable to cells that had not been LPS activated.

We also studied if IL-10 activation was affected by E7-microparticles, on the basis that IL-10 down-regulates pro-inflammatory cytokines and inhibits generation of CD8 effector T cells^31^. IL-10 expression is increased following LPS treatment, through sequential induction of type I IFN and IL-27^32^. We incubated LCs with E7 or control microparticles and investigated whether the treated cells expressed IL-10 following LPS activation. We found that the number of IL-10 positive cells was only modestly increased following activation with LPS, and in contrast to what we observed with IL-12 p40, E7-MP did not have any significant impact on IL-10 expression (Fig. 6). From these results we conclude that LCs are not fully activated in the presence of E7 MPs, with the consequence that IL-12 p40 subunit production is inhibited.

## Discussion

In this study we report that expression of HPV16 E7 increased microparticle production from murine and human keratinocytes, and that co-culture of LCs and T cells with those microparticles suppresses antigen-specific cytotoxicity. A large body of evidence has demonstrated the importance of CD8 T cell cytotoxicity in the elimination of virally infected cells (reviewed in Lechner, *et al*.^33^, and destruction of tumours, including cervical cancer^34–36^. If these data were to translate to HPV infection, suppression of the cytotoxic response would likely contribute to evasion of immunity by the HPV infected or transformed cells. We also report here that expression of the IL-12 p40 subunit is strongly suppressed by E7 microparticles and that CD40 expression is reduced. As CD40 licenses APC to prime T cells, and IL-12 promotes clonal expansion of T cells, the regulation of these proteins in the presence of E7-microparticles is the likely mechanism whereby the CD8 T cell response to the co-expressed OVA is being impaired.

LCs have the capacity to be activated, and to activate T cells in the epidermis^11^. In this study, LCs were activated by LPS *in vitro*, LCs from epidermal sheets were competent to migrate from the epidermis, and LCs exposed to control microparticles and OVA provoked an OVA-specific cytotoxic response. In addition, we and others have shown that cytotoxic T cell responses can be generated to skin-expressed OVA *in vivo*^37,38^. There is now also substantial evidence supporting the immune suppressive effects of E7. Consistent with the suppression by E7 observed here, we also previously have shown that the cytotoxic T cell response to OVA was suppressed *in vivo* when E7 was co-expressed in the OVA-expressing epidermal keratinocytes^37^. Furthermore, E7 transgenic skin grafted onto immune competent E7-immunised recipient mice is not rejected^39^, an immune suppressive environment is created following mast cell infiltration in the HPV16 E7 skin-expressing transgenic mouse^40^, and surface MHC-I is down-regulated on cells expressing E7^41^. The immune suppressive effects of E7 microparticles *in vitro* suggests a role for E7 in the regulation of antigen presentation by resident LCs, with consequential impaired signaling to CD8 T cells and defective development of effector CTLs, and adds to a number of immune regulatory effects reported to occur in HPV16 E7-expressing mouse skin.

There is evidence of regulation of LCs in HPV16 infection. Others have reported suppression of LC activation following uptake of HPV16 virus-like particles (generated from the L1 and L2 capsid proteins of the virus)^42^. We have previously shown that LC numbers are reduced in the epidermis of HPV16-infected cervical lesions, which is associated with reduced expression of E-cadherin on the infected keratinocytes^14,43^. As E-cadherin on keratinocytes binds to E-cadherin on LCs, it was plausible that E-cadherin expression on LCs would be altered when co-cultured with E7-microparticles, however when we tested this here in the mouse it was not the case. The biological significance of LC regulation in human infection remains to be elucidated, particularly as LCs are not essential for a CD8 T cell response to skin-expressed OVA in the mouse^37^. Similarly, we show here that microparticles are produced from HPV16 E7 expressing human-derived keratinocytes, and from HPV16 E6 and E7 expressing murine keratinocytes, however expression of microparticles from transformed cell lines was more variable and was also likely to be regulated by other cellular proteins. An analysis microparticle production from cervical lesions from women with different grades of CIN from persistent or regressing lesions is required to establish the relevance of our observations to human infection, high grade neoplasia and cervical cancer.

We found that co-culture of LCs with E7 microparticles suppressed the increased CD40 expression that normally occurs on LCs following LPS treatment. LPS triggers MyD88 dependent and independent signaling pathways in LCs through Toll like receptor 4 (TLR4). The MyD88 dependent pathway activates genes encoding surface co-stimulatory molecules such as CD40 and CD86 (reviewed in^44^). LPS activation through TLR4 induces recruitment of NF-κB p65, p50 and STAT-1a to the CD40 promoter, as well as modifying histones H3 and H4 at the promoter to induce CD40 expression^45^. E7 is known to inhibit NF-κB^46^, and interference of the NFκB pathway by HPV has been reported previously in keratinocytes by HPV-induced expression of the cellular protein ubiquitin carboxyl-terminal hydrolase L1 (UCHL1)^47^. Proteins from other pathogens modulate CD40 through NF-κB, and this may be occurring here following LC uptake of E7-microparticles following their fusion or through internalization by pinocytosis or phagocytosis^48,49^. For example, Human T cell Leukemia Virus activates expression of CD40 gene by interacting with NF-κB through the viral protein Tax^50^, and adenylate cyclase toxin from Bordetella pertussis toxin^51^ inhibits NF-κB activation and costimulatory molecule expression.

In this study CD40 activation was suppressed in the in the presence of E7-microparticles, whereas CD86 was unaffected. Although upregulation of CD80 and CD86 typically follows CD40 expression and recruitment of TNF Receptor Associated Factor family of proteins (TRAF) to its cytoplasmic tail^52^, induction of CD86 in the absence of an increase in CD40 expression also can occur. Exposure to zwitterionic polysaccharides induces expression of CD80 and CD86 on antigen presenting cells, in the absence of any increase in CD40^53^.

The NFκB pathway is also significant in the regulation of LPS induced IL-12 p40^54^. When we investigated regulation of IL-12 p40 expression by LCs exposed to E7 microparticles following LPS activation, we found that virtually none of the LCs co-cultured with E7-microparticles expressed IL-12 p40 following LPS activation. This was a profound inhibitory effect that was not observed with the control microparticles. IL-12 is integral to the induction of CD8 differentiation^55,56^, and is a key determinant of whether immunity or tolerance is induced to a given antigen. The impaired IL-12 p40 expression by LCs co-cultured with the E7 microparticles may translate into reduced expression of secreted IL-12, which would likely impact on the efficacy of the CTL response.

Expression of IL-12 and the anti-inflammatory IL-10 are typically inversely regulated^57^, however IL-10 expression was not increased in LCs exposed to E7-microparticles as might be expected. The lack of difference in IL-10 expression induced by E7-microparticles compared with controls may be a result of temporal differences in peak expression between these cytokines, or may reflect a more generalised suppression of cytokine expression by E7-microparticles.

As far as we are aware, regulation of CD40 and IL-12p40 by microparticles from HPV16 oncoprotein expressing cells has not previously been reported; indeed the only role previously attributed to microparticles in relation to HPV has been in mediating HPV6 and 11 infection of macrophages^58^. Viral suppression of the cytotoxic T cell response is a likely contributing factor to HPV persistence, and the evidence here suggests that microparticles may be shed from HPV infected cells and that those microparticles may suppress antigen presenting cell function and consequential T cell activation. Because they are shed, they may exert their effects beyond the local microenvironment, conceivably on dermal dendritic cells and also in the local draining lymph nodes. Additionally, it is of significance that these microparticles can mediate effects without a requirement for viral infection of the target cells. This may be of particular relevance for a virus such as HPV, with its specific tropism for keratinocytes. It is of interest to consider the immune regulatory effects of E7-microparticles in the context of the development of drugs to inhibit their production, and the potential therapeutic benefits that those drugs may have in the resolution of persistent HPV infection and any consequential cancer that may arise.

## Methods

### Ethics

All animal research was carried out with ethical approval (AEC12/14) from the University of Otago Animal Ethics Committee, and in accordance with New Zealand Animal Welfare Act (1999) and National Animal Ethics Committee (Ministry of Primary Industries, NZ) guidelines. All genetic modifications were carried out under approval by the Environmental Protection Authority (New Zealand).

### Cells

293 TT (obtained from J. Schiller, NIH, USA), a human embryonic kidney cell line containing an extra copy of the SV40 large T antigen, was used for the generation of lentiviral vectors^59^. Human HaCaT (originally obtained from N Fusenig, DKFZ, Heidelberg, Germany)^60^ and mouse (C57BL/6) PDV (obtained from A. Cano, University of Madrid, Spain)^61^ immortalized keratinocyte cell lines were also used in this study. All cell lines were grown in Dulbecco’s Modified Eagle Medium (DMEM, Sigma Aldrich) with 10% fetal bovine serum (FBS) and 0.1 mg/ml penicillin/streptomycin (Roche Molecular Biochemicals). Cells were incubated at 37 °C and 5% CO_2_.

### Lentivirus Production

The plasmids pMD2.G, encoding vesicular stomatitis virus glycoprotein (Addgene, USA), pMDLg/pRRE encoding Gag-pol (Addgene, USA) and pRSV-REV, encoding Rev (Addgene, USA) and the protocol developed by Tiscornia, *et al*.^62^ were used for this study. The lentivirus expression plasmid pSMPUW-IRES-Puro (Cell Biolabs, CA), expressing the gene of interest under an EF-1 promoter, and containing an IRES and puromycin resistance gene following multiple cloning sites, was used for this research. A plasmid containing HPV16 E7 tagged with HA on the 5′ end (pSMPUW-HAE7) was constructed. 293 TT cells were seeded at a density of 2.5 × 10^6^ cells in a T25 tissue culture flask and incubated at 37 °C, 5% CO_2_ overnight. The lentiviral vectors containing HAE7 or no insert were generated by transfection of 293 TT cells with PEI, equal parts of pMD2.G, pMDLg/pRRE and pRSV-REV, and two-fold of pSMPUW-HAE7 or pSMPUW. Supernatants were harvested 48 h and 72 h post-transfection and combined, filtered through a 0.45 µm filter, then centrifuged at 70,000 × *g* for 2 h at 20 °C. Viral pellets were resuspended in 50 mM Tris pH 7.5, 140 mM NaCl, 5 mM EDTA, divided into aliquots and frozen at −20 °C until required.

### Generation of HPV16 HAE7 and control cell lines

Target HaCaT and PDV cells were seeded at 1 × 10^5^ cells per well in cDMEM in a 6 well plate. After 24 h, media was replaced with 500 µl of lentivirus and 500 µl cDMEM. Media was changed the following day and remained on cells for a further 24 h. HaCaT cells were then selected with 1 μg/ml puromycin (Gibco, Invitrogen), while PDV cells were selected with 1.1 μg/ml puromycin (54), chosen based on the results of a puromycin kill curve. Surviving cells were expanded and used to generate microparticles.

### Microparticle Harvest and Quantification

Microparticles were purified as previously described^23,63^. Briefly, 1 × 10^6^ HaCaT or PDV cells were seeded and incubated overnight. Supernatants were collected from confluent cells, and centrifuged at 1500 × *g* for 5 min to remove cells and cellular debris. The supernatants were then centrifuged at 17,000 × *g* for 20 min at 4 °C to pellet the microparticles.

Microparticles were quantified using flow cytometry. Purified microparticles were resuspended in annexin binding buffer (10 mM HEPES, 140 mM NaCl and 2.5 mM CaCl_2_) and stained with 1.25 µl annexin V Alexa Fluor 488 (Invitrogen), in order to detect phosphatidylserine, then analysed using flow cytometry. The sizing gate (0.1–1 μm) was defined using spherical beads ranging from 0.7–7.4 µm (Spherotech) that were added to the sample. In addition to size, microparticles were distinguished by the expression of phosphatidylserine on the outer surface (positive annexin V staining). The absolute number of microparticles in each sample was determined using a defined number of AccuCount fluorescent particles (Spherotech). All analysis of flow cytometry data was carried out using FlowJo vX software.

### Generation of Langerhans cells

LCs were differentiated from murine red blood cell-depleted bone marrow precursor cells^64^ obtained from C57BL/6 mice (HTRU Animal Breeding Facility, University of Otago, NZ) by culturing 1 × 10^6^ cells/well of a six-well tissue culture dish in 1 ml complete DMEM supplemented with 10 ng/ml GM-CSF, M-CSF (BioLegend, CA) and TGF-β (R&D Systems, MN). Cells were cultured for a total of four days, with medium being replaced at day 1 and day 3. Cells were confirmed by flow cytometric analysis to have a phenotype consistent with what has been previously reported, i.e. CD11c, MHCII and EpCAM positive.

### Microparticle treatment of cells and functional analysis

Microparticles purified from supernatants from E7-expressing or control E7rev PDV or HaCaT cells were applied to LCs at a ratio of 5:1. Cells were incubated for 48 h, and in experiments where cells were stimulated with lipopolysaccharides from Escherichia coli 055:B5 (LPS; Sigma L2880), 100 ng/ml LPS was added to cultures for a further 24 h. In experiments where intracellular staining was carried out, 1 μg/ml of Brefeldin A was added to the cells 4 h before harvest.

Cells were stained using fluorescent antibodies against the following antigens from BioLegend, catalogue numbers in brackets: Alexa Fluor 647 anti-mouse EpCAM (118212); APC anti-mouse IL-12/IL23 p40 (505205); Brilliant Violet 421 anti-mouse CD11c (117330); PE/Cy7 anti-mouse CD40 (124622); PE/Cy7 anti-mouse CD86 (105014); FITC anti-mouse I-A/I-E MHCII (107605). Fluorescent antibodies against the following antigens from BD Biosciences were used: anti-mouse IL-10 (554467); 488 anti-mouse E-cadherin (560061). Live/dead distinction was carried out following the addition of Zombie NIR dye to cells (BioLegend; 77184). Compensations were set using One Comp Beads stained with the relevant fluorescent antibodies. In all cases fluorescence minus one (FMO) controls were used to set gates, to ensure that spectral overlap between antibodies was fully compensated and Fc block was used in all experiments.

Cell staining was carried out using well-established protocols. Briefly, 1 × 10^6^ cells per tube were re-suspended in 100 µl of PBS and Zombie NIR dye and incubated for 15 min at RT in the dark, followed by a 2 min. incubation with Fc block. Surface-marker staining antibodies were then added to cells and incubated for 15 min. in the dark on ice. Cells were washed in PBS containing 0.01% sodium azide and 0.1% BSA (FACS buffer), and resuspended in FACS buffer prior to flow cytometric analysis.

For intracellular staining, cells were fixed in 4% paraformaldehyde for 30 min on ice, washed twice in Cell-Perm buffer 1 (0.1% saponin, 0.1% BSA 10 mM HEPES in PBS) then re-suspended in 100 µl of the buffer and incubated on ice for 30 min. IL-12 and IL-10 specific fluorescent antibodies were added to the cells and incubated a further 30 min. Cells were washed twice with Cell-Perm buffer 2 (0.1% saponin, 2% BSA 10 mM HEPES in PBS) and twice with FACS buffer. Finally, the cells were re-suspended in 200 µl FACS buffer for flow cytometry analysis. The analysis was performed using FlowJo vX software.

### Western blot

1 × 10^7^ of E7-PDV and control cells were harvested, re-suspended in 800 µl of ice cold cell lysis buffer (Tris-buffered saline, pH 7.5 containing sodium deoxycholate, Triton X100 and protease inhibitors), and incubated for 30 min on ice. Cellular debris was separated by centrifugation at 1500 × *g* for 5 min at 4 °C. The supernatant was stored at −20 °C until used. Immuno-precipitation (IP) of HPV16 E7 was carried out. The lysate was pre-cleared by incubating with Protein G PLUS-Agarose (Santa Cruz Biotechnology, Texas, USA), then incubated at 4 °C O/N with mouse anti-haemagglutinin (HA) (B9183; Sigma, Missouri, USA) washed protein G agarose beads. Beads were washed, resuspended in sample buffer and boiled for 5 min. The supernatant was loaded onto 5–15% gradient SDS-PAGE gel, the proteins transferred to nitrocellulose membrane and the HA-tagged protein was detected using primary anti-HA-Biotin (Sigma-Aldrich, MO, USA), and secondary Streptavidin 680RD IRDye® (LI-COR Biosciences, NE, USA).

### LC migration from skin explant cultures

Skin explant culture was performed as previously described (63). Briefly, C57/BL6 mice (sourced as above) were euthanised and their ears were removed at the base, sterilized in 70% ethanol and air-dried. Ears were then split into dorsal and ventral halves using forceps. The dorsal halves of the ears (both dermis and epidermis) were then incubated in Iscove’s medium (Sigma Aldrich) with 0.1% 2-mercaptoethanol, 10% FCS and 0.1 mg/ml penicillin-streptomycin (complete Iscove’s medium) in 24-well plates with one ear per well. Ears were left untreated or treated with 5000 microparticles/well generated from PDV cells, PDV cells transduced with E7rev or PDV cells transduced with HPV16E7. After 48 h of culture, the ears were removed and cells that had migrated out of the explant were collected and counted. The experiment was also repeated with equal volumes (100 µl) of microparticle preparation from the different transduced cell lines in order to account for differences in the number of microparticles produced by cells expressing HPV16E7.

### *In vitro* cytotoxicity assay

Day 4 LCs generated as described above were harvested, the CD11c+ EpCAM+ cell content determined by flow cytometric analysis and 1 × 10^6^ CD11c+ cells/mL were incubated with or without 1 µg/mL SIINFEKL for 2 h at 37 °C. CD8+ OTI T cells were isolated from a red blood cell depleted spleen cell suspension obtained from OTI transgenic mice (HTRU Animal Breeding Facility, University of Otago, NZ) using Miltenyi MACs immune-separation as per the manufacturer’s instructions, seeded at 2 × 10^6^ cells/ml with washed, peptide-pulsed or unpulsed BMDC at 2 × 10^5^ cells/ml in complete DMEM and incubated for 48 h at 37 °C. Target cells were prepared from red blood cell-depleted spleen cells obtained from C57BL/6 mice (sourced as above) and either pulsed with SIINFEKL for 2 hrs at 37 °C, or left unpulsed. The washed cells were then incubated with carboxyfluorescein succinimidyl ester (CFSE) at either a high concentration (final concentration of 1:8000 of a 10 mM CFSE stock in DMSO) for peptide-pulsed cells or a low concentration (final concentration of 1:64,000 of a 10 mM CFSE stock in DMSO) for unpulsed cells for 8 min at RT, washed and re-suspended at 2 × 10^6^ cells/ml. The two suspensions were mixed at a 1:1 ratio, added to non-adherent CD8+ OTI cells that had been removed from adherent LCs in varying ratios: 1:3, 1:1 and 3:1 and incubated for 4 h at 37 °C. The remaining CFSE positive target cells were then quantified using flow cytometry. Peptide-specific cytotoxicity (% specific lysis) was calculated as 1 – ratio unpulsed/ratio pulsed × 100. The ratio = % CFSE^lo^ splenocytes/% CFSE^hi^ splenocytes.

## Electronic supplementary material

Supplementary Information
