# Supplementary material for: Microparticles produced by human papillomavirus type 16 E7-expressing cells impair antigen presenting cell function and the cytotoxic T cell response
Source: Sci Rep. 2018 Feb 5;8:2373. doi: 10.1038/s41598-018-20779-2 (PMC5799164; doi:10.1038/s41598-018-20779-2)
Supplement: Supplementary file 1 — Supplementary Information [file 41598_2018_20779_MOESM1_ESM.docx]

**Microparticles produced by human papillomavirus type 16 E7-expressing cells impair antigen presenting cell function and the cytotoxic T cell response**

Zhang, J., Burn, C., Young, K., Wilson, M., Ly, K., Budhwani, M., Tschirley, A., Braithwaite, A., Baird, M. and Hibma, M.

Department of Pathology, Dunedin School of Medicine, University of Otago, Dunedin, New Zealand

Correspondence to: Merilyn Hibma

Department of Pathology, Dunedin School of Medicine, University of Otago, P O Box 56, Dunedin 9054, New Zealand

Tel: +643 479 7726

E-mail: merilyn.hibma@otago.ac.nz

Running title: HPV E7 immune-suppressive microparticles

Keywords: Human papillomavirus; HPV16; E7; microparticle; microvesicle; immune suppression; T cell response; CD40; IL-12.

**
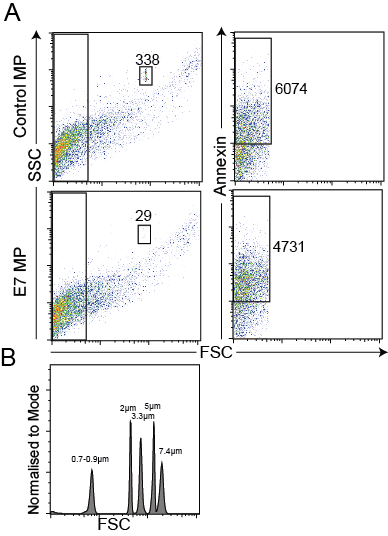
**

**Supplementary figure 1.** Flow cytometric analysis and quantification of microparticles from HaCaT E7 and control cells. Representative dot plots of the approximately 10,000 events collected for each sample are shown. (A) The left-hand panels show the forward and size scatter gating of microparticles HaCaT E7 and control cells. The absolute number of microparticles was determined using a defined number of 5.2 µm counting beads (equivalent numbers of beads were added to each sample), indicated in the upper right box in the forward and side scatter plots. The right-hand panels indicate the annexin positive events (i.e. phosphatidylserine positive events), with the gate set using unstained control samples. (B) Histogram of the sizing beads of sizes as indicated.

**Supplementary figure 2.** Characterisation of bone marrow differentiated LCs and their comparison to bone marrow differentiated DCs. Bone marrow derived and differentiated cells were harvested and stained with antibodies specific for DCs and LCs. (A) Live, single cells that were MHCII and CD11c positive were gated and analysed for EpCAM, CD40, CD86 and E-cadherin (B). Consistent with the reports of others, cells differentiated to LCs were EpCAM positive, whereas cells differentiated to DCs were not.


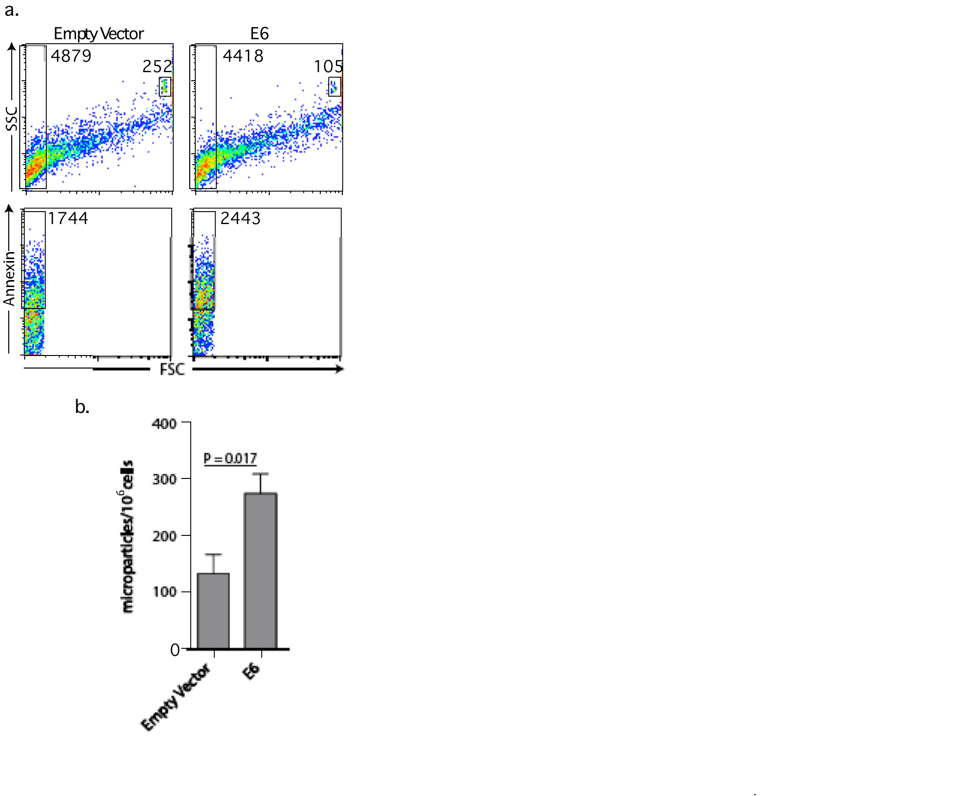


**Supplementary figure 3.** Quantification of microparticles in E6-expressing PDV cells. PDV cells transduced with lentivirus produced from the pSMPUW plasmid containing a flag-tagged E6 and then selected with puromycin. Microparticles were harvested from the HPV16 E6 expressing PDV cells or PDV cells transduced with lentivirus produced from the pSMPUW cells with no insert after 24h in culture. **a.** The absolute number of microparticles was determined using a defined number of 5.2µm counting beads, indicated in the upper right box in the forward and side scatter plots, and the size gate for microparticles was set using 0.7-0.9µm sizing beads. The right-hand panels indicate the annexin positive events (phosphatidylserine positive), with the gate set using the unstained control samples. **b.** The quantification of microparticles from PDV and PDV E6 cells (n = 3 independent experiments; mean ± standard error of the mean is shown; Statistical analysis was carried out using an unpaired Welch’s t-test).
